# Quantitive Assessment of Gustatory Function and Its Association with Demographics, and Systemic Morbidity

**DOI:** 10.3390/biology13010050

**Published:** 2024-01-18

**Authors:** Doron J. Aframian, Alaa Zedan, Weaam Agbariah, Andra Rettman, Galit Almoznino

**Affiliations:** 1 Sjögren’s Syndrome Center, Hadassah Medical Center, Jerusalem 9112001, Israel; 2 Faculty of Dental Medicine, Hebrew University of Jerusalem, Jerusalem 9112001, Israel; 3 Department of Oral Medicine, Sedation & Imaging, Hadassah Medical Center, Jerusalem 9112001, Israel; 4 Big Biomedical Data Research Laboratory, Dean’s Office, Hadassah Medical Center, Jerusalem 9112001, Israel

**Keywords:** taste/taste, gustatory dysfunction, hypogeusia, ageusia, taste strips test, pathophysiology, oral diagnosis, oral medicine, oral-systemic disease(s), risk factor(s), systemic health/disease

## Abstract

**Simple Summary:**

This study analyzed the associations of taste dysfunctions as measured by validated taste strips with demographics and co-morbidities. The study demonstrated that taste dysfunction was associated with older age, male sex, and co-morbidities such a previous major trauma, being under chemotherapy, zinc deficiency, burning mouth syndrome, and exposure to toxins. The study highlights the importance of assessment of taste disorders with a validated objective tool of taste strips and of comprehensive assessment of co-morbidities as part of the diagnostic process of patients with subjective complaints of taste disorders.

**Abstract:**

This study aimed to analyze the associations of gustatory dysfunction as measured by validated taste strips with demographics and co-morbidities. This cross-sectional study retrospectively analyzed records of patients who attended the Orofacial Chemosensory Center of Hadassah Medical Center between 2017 and 2020. Taste strips were used as a validated method to determine taste dysfunction. A total of 272 subjects were included, 137 (50.4%) women and 135 (49.6%) men, with a mean age of 53.5 ± 19.3 years and age range of 18–98 years. The total taste score among the study population was 8.53 ± 4.03 (scale range 0–16). Age had a significant negative correlation with the total taste score (*p* = 0.001), and men exhibited worse total (*p* < 0.001), salty (*p* = 0.003), and bitter (*p* < 0.001) scores. Major trauma was associated with worse total (*p* < 0.001) and specialized taste assessments (sweet (*p* = 0.001), sour (*p* = 0.002), salty (*p* = 0.016), and bitter (*p* < 0.001)). Chemotherapy was associated with reduced total (*p* < 0.001), salty (*p* = 0.003), and bitter (*p* = 0.001) taste scores. Zinc deficiency exhibited worse salty (*p* = 0.027) and total (*p* = 0.038) taste scores. Patients with burning mouth syndrome (BMS) showed higher salty scores (*p* = 0.017). Patients who experienced exposure to toxic chemicals exhibited worse salty scores (*p* = 0.024). We conclude that gustatory dysfunction is associated with older age, male sex, and co-morbidities of major trauma, current chemotherapy, zinc deficiency, BMS, and exposure to toxins. The study highlights the importance of systemic evaluation and quantitive gustatory dysfunction assessment as part of the diagnostic process of patients with subjective complaints of taste disorders.

## 1. Introduction

The human sensory system discerns five tastes, namely, sweet, sour, bitter, salty, and umami [1], and there are many other sensory systems in human bodies. Taste receptors reside in taste buds, averaging approximately 7500 in number and situated in various regions in the oral cavity, larynx, and pharynx. The taste buds exhibit multiple and spatially segregated neural innervations, affording considerable redundancy or capacity in the event of injury or impairment to any specific nerve or subset thereof [2].

Taste disorders include *Ageusia*, the inability to perceive tastes through the taste receptors, whereas *Hypogeusia* signifies a diminished capacity for taste sensation. *Hypergeusia* refers to an intensified sensitivity to one or more tastants, typically characterized by lower threshold values. *Dysgeusia* encompasses any form of distorted taste perception, with the presence of a stimulus giving rise to stimulated dysgeusia, while unprovoked sensations are termed *Unstimulated dysgeusia* or *Phantogeusia* [3].

Taste dysfunction can adversely affect food intake leading to weight loss, malnutrition, and decreased quality of life [1,2]. Taste disorders can arise from peripheral or central pathology, often exhibiting overlapping characteristics. Peripheral conditions include infection, neuropathy, neoplasia, surgery, and trauma, while central processes involve brain stem, thalamic, and cortical disorders; traumatic brain injury; strokes and tumors; epilepsy; peripheral facial palsy; multiple sclerosis; and migraines [3]. Peripheral facial palsy can cause gustatory dysfunctions in the anterior two-thirds of the tongue due to the transmission of taste sensations via the chorda tympani nerve and geniculate ganglion to the nucleus tractus solitarius in the medulla oblongata [4]. Additionally, certain conditions do not easily fit into either category and encompass immune-related diseases, metabolic and endocrine disorders, medications, toxins, pollutants, local oral disorders, insufficient saliva, and gastroesophageal reflux disease [5] Additionally, prevalent medical disorders like hypothyroidism, diabetes, chronic liver disease, kidney disease, and immune system diseases contribute to taste disorders [1,6,7,8]. Neurological disorders such as Bell’s palsy, multiple sclerosis, certain brain stem strokes, and brain tumors also impact taste perception [1]. Gustatory dysfunctions can also be induced by olfactory disorders, as evidenced by the research conducted by Hernandez et al., illustrating that chemosensory functions (orthonasal olfactory, trigeminal, retronasal olfactory, and gustatory) and nasal airflow are correlated with each other [9]. The incidence of smell and taste disorders has significantly increased since the coronavirus disease 2019 (COVID-19) pandemic due to the fact that smell and taste disorders are complications of severe acute respiratory syndrome coronavirus-2 (SARS-CoV-2) infection [10]. Treatment is focused on addressing the underlying cause [11]. Many taste disorders resolve spontaneously within a few years of onset, but immediate interventions can be implemented [12]. Zinc salts are predominantly used for *ageusia*, primarily in cases of proven deficiency like those related to renal or liver disease [12]. Herbal agents and natural products have made a significant entry into oral care products in recent years, complementing traditional treatment procedures due to their physicochemical and therapeutic properties [13].

Accurate diagnosis of taste disorders necessitates the implementation of taste measurement techniques. Nevertheless, human self-assessment of taste dysfunction, especially when it is not complete, is known for its inherent inaccuracy [2,14]. In many cases, no or only a slight correlation can be observed between objective testing and the subjective reports of the patients. For example, Nørgaard et al. demonstrated that subjective gustatory dysfunction was poorly correlated with measured gustatory dysfunction [15]. It had been suggested that subjective gustatory dysfunction underestimates objective dysfunction; therefore, it was recommended that, in particular, older men with diminished olfactory function should undergo gustatory function testing regardless of their self-reported gustatory function status [16]. To overcome this limitation, validated tests have been developed as highly sensitive means of assessing taste recognition thresholds in humans [17].

Considering the importance of addressing demographics as well as the underlying co-morbidities, and the importance of employment of validated taste measurement, it seems crucial to understand the impact of different co-morbidities on taste scores. Therefore, the main objectives of the study are as follows:

1. To describe the demographic and medical profiles of the patients attending the Orofacial Chemosensory Center clinic examined for taste disturbances.
2. To analyze the associations of gustatory function as measured by validated taste strips with demographics and co-morbidities, and thus identify specific patient demographics and co-morbidities that are associated with quantitative gustatory dysfunction.

The study hypothesized that gustatory dysfunction scores measured by validated taste strips would correlate with the presence of specific patient demographics and certain co-morbidities.

## 2. Materials and Methods

### 2.1. Study Design

This cross-sectional, records-based study included a retrospective analysis of the entire population of patients who were examined for taste disorders at the Center for Taste and Smell Disorders, Hadassah Medical Center.

### 2.2. Ethical Approval

The study adheres to the STROBE guidelines, and approval for the study was obtained from the Hadassah Medical Center Institutional Review Board (IRB number: HMO-0005-22). Since this retrospective study only included analysis of anonymous medical records, the IRB gave an exemption from written informed consent.

### 2.3. Eligibility Criteria

Inclusion criteria: Patients examined for taste disorders at the center between 2017 and 2020, aged ≥ 18.

Exclusion criteria: Pregnancy or lactation, lack of data in the medical file, positive COVID-19 during the examination, and post-COVID-19 taste dysfunction; the latter were excluded to focus on the impact of demographics and systemic co-morbidities and not on COVID-19 taste dysfunction.

### 2.4. Collected Data and Definition of the Variables

Data were collected from the medical records and included the following parameters: sex, age, type of taste disorder, and co-morbid medical illnesses. These are described in the following subsections.

#### 2.4.1. Dependent Variable: Taste Scores

The taste scores are continuous variables measured under the assessment of gustatory function, utilizing the validated “Taste Strips Test” [18]. All Taste Strips tests were performed by a single experienced oral medicine specialist (G.A). Before testing, participants were instructed to refrain from consuming food, smoking, or using chewing gum for at least one hour and only allowed to drink water. The test involved the sequential presentation of taste strips in a pseudo-randomized order. The complete set comprised 16 containers with four concentrations each of sweet, sour, salty, and bitter tastes, along with three blank strip containers. The taste strips were positioned in the middle of the anterior third of the extended tongue, with the mouth closed and the tongue moving slowly [18]. Patients were required to report any taste perception and identify the specific taste. Patients were provided with 5 options for selecting the correct taste: sweet, sour, salty, bitter, and no taste (for the blank strips). Scoring was based on correct identifications, with each accurate answer earning one point. If all 16 strips are identified correctly, the maximum possible score is 16 (4 correct for each taste quality). Blanks are not counted during evaluation.

*Normoguesia* was defined for scores ≥ 9 [19]. For sweet, sour, and salty tastes, scores ≥ 2 were indicative of Normoguesia, while a score ≥ 1 indicated Normoguesia for bitter taste [19]. Patients with hypogeusia (identification < 9) might exhibit ageusia for specific taste qualities, such as hypogeusia with a total score of 7 accompanied by ageusia for sour taste [18,19]. Assessment of dysgeusia and phantogeusia was based on subjective questions (yes/no). Hyposalivation was detected on clinical examination, revealing one of the following: the oral mucosa and a gingiva appear bright, pale, or atrophic; the dental mirror adheres to the oral mucosa or tongue, revealing an absence of saliva accumulation on the floor of the mouth.

#### 2.4.2. Independent Variables

The independent variables included demographics and co-morbidities. All co-morbidities were based either on the medical information summary of referral letters from the general physician of the patient or on the findings of the work-up in our Taste and Smell clinic.

1. Demographics and smoking status: age-quantitative continuous, sex-male/female, and current smoker: yes/no.
2. History of head and/or neck trauma: Categorized into two types—minor trauma, e.g., injuries resulting from invasive or prolonged dental procedures, and major trauma including falls, road traffic accidents, and altercations. The history of the surgical procedure was recorded.
3. Local conditions: xerostomia, hyposalivation, and burning mouth syndrome (BMS).
4. Zinc, B-12, and iron deficiencies.
5. Exposures: medications, toxins, chemotherapy, and radiation.
6. Systemic diseases: yes/no.

### 2.5. Statistical Methods

Data were analyzed using IBM SPSS (Statistical Package for the Social Sciences) software version 28.0 (Chicago, IL, USA), and a *p*-value of <0.05 was considered statistically significant.

Descriptive statistics: Continuous variables are presented using means and standard deviations. The categorical variables are presented as percentages and frequencies.

Univariate analyses: The associations between the taste scores and categorial parameters were examined using an independent *t*-test or ANOVA, and the associations with continuous variables were tested using Pearson’s correlation. Tests to assess normality included skewness and kurtosis. The univariate analysis employed non-parametric Independent-Samples Mann–Whitney U Test, Kruskal–Wallis test, and Spearman’s correlation test; however, since there were no differences between the parametric and non-parametric results, we show the results of the parametric tests.

Procedure to decrease the false discovery rate (FDR): Following the univariate analysis, we applied the Benjamini–Hochberg (BH) procedure to decrease the false discovery rate (FDR). The criteria to enter the multivariate analysis were independent variables that had significance in the univariate analysis that remained statistically significant following the BH correction.

Multivariate analysis with collinearity statistics: Multivariate linear regression analysis with the dependent variable including collinearity statistics was conducted. The variance inflation factors (VIFs), which are 1/tolerance, were calculated. While VIF < 10 is usually considered indicative of collinearity, in weaker models, VIF > 2.5 may be a cause for concern; therefore, the current study used VIF < 2.5 as a cutoff. In the case of highly correlated variables, only one was used in the multivariate model, and the context determined which variable would be used in the model.

## 3. Results

### 3.1. Demographics and Clinical Parameters of the Study Population

The study included 272 patients. Table 1 presents the demographics, smoking status, and co-morbidities of the study population. The mean age of the study population was 53.56 ± 19.27 years, median 56 years, and range 18–98 years. There were 137 (50.4%) women and 135 (49.6%) were men. A total 42 patients (15.4%) were current smokers, 48 (17.6%) patients suffered major trauma, and 16 (5.9%) had minor trauma. Xerostomia was reported by 37 (13.6%) patients, and objective hyposalivation was denoted by 28 (10.3%) patients. According to subjective questions, 53 subjects (19.5%) reported on dysgeusia and 34 subjects (12.5%) reported on phantogeusia. Subjective olfactory complaints were as follows: anosmia (78 patients, 28.6%), hyposmia (63 patients, 23.1%), and phantosmia (5, 1.8%). The most common diseases were hypertension (43, 15.8%); diabetes mellitus (37, 13.6%); upper respiratory infection (37, 13.6%); gastrointestinal disease (35, 12.9%); neurologic disease, e.g., Parkinson’s (23, 8.5%); and liver disease, e.g., liver cirrhosis (2, 0.7%) (Table 1).

### 3.2. Total and Specific Taste Scores (Sweet, Salty, Sour, Bitter) of the Study Population

**Figure 1:**
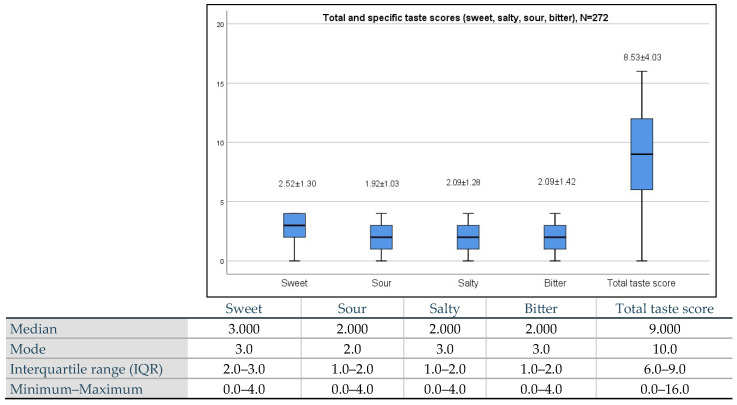
The mean and standard deviations of the total and specific taste scores (sweet, salty, sour, and bitter) of the study population.

Figure 1 presents the mean total taste scores and the specific taste scores (sweet, salty, sour, and bitter) of the study population. The total taste score among the study population was 8.53 ± 4.03 (Normoguesia ≥ 9). Sweet exhibited the highest (i.e., better) specific taste score (2.52 ± 4.03), followed by salty and bitter scores (2.09 ± 1.28 and 2.09 ± 1.42, respectively), and the lowest score was sour (1.92 ± 1.03).

### 3.3. Univariate Analyses of the Associations of Demographics and Co-Morbidities with the Specific Taste and the Total Taste Scores

Table 2 presents the analysis of the statistically significant associations of demographics and co-morbidities with the specific taste and the total taste scores. Univariate analysis with non-parametric showed no differences between the parametric and non-parametric results (see Supplementary File); therefore, we show the results of the parametric tests in Table 2. The following demographic and clinical parameters were statistically significantly associated with several recognition taste scores (Table 2):

1. Age had a statistically significant slight negative correlation with sour and bitter special taste scores as well as with the total taste score since the correlation coefficients were neglectable (<0.3).
2. Men, patients with a history of major trauma, and current chemotherapy treatment were independently associated with lower (i.e., worse) taste scores in all specialized as well as total taste scores. Minor trauma was not associated with worse taste scores.
3. Patients with zinc deficiency exhibited worse sweet, salty, and total taste scores compared with those without the deficiency.
4. BMS was associated with higher salty and total taste scores.
5. S/P upper respiratory tract infection (S/P URTI) was associated with higher taste scores in all specialized as well as total taste scores.

The following parameters were associated only with specific taste recognition scores:

Specialized sweet scores. Patients with hyposalivation and autoimmune disease exhibited worse (i.e., lower) sweet scores.

Specialized salty scores. Patients who were exposed to toxic chemicals exhibited worse salty recognition scores compared with those without such exposure. Patients with gastrointestinal disease exhibited better salty recognition scores compared with those without the disease.

Specialized bitter scores. Patients with kidney disease and obesity exhibited worse bitter taste scores.

There were no statistically significant associations between smoking status and the other co-morbidities presented in Table 2 with the taste scores.

### 3.4. Benjamini–Hochberg (BH) Procedure to Decrease the False Detection Rate (FDR)

Following the univariate analysis, we performed the Benjamini–Hochberg (BH) procedure to decrease the FDR, and the results are shown in Table 3. Ensuring the effective management of type I errors during multiple hypothesis testing holds significant significance in the realm of biomedical research. Consequently, subsequent to conducting multivariate analysis, we implemented the BH procedure for its ability to strike a nuanced equilibrium between controlling the FDR and preserving statistical power. The practical significance of the BH procedure is that only variables that were “significant” in the BH procedure entered the multivariate analysis in the next step, while those with “Not Significant” test results were not included in subsequent multivariate analyses.

### 3.5. Multivariate Analysis of Specific and Total Taste Scores with Statistically Significant Independent Variables

Following the BH procedure, we conducted a multivariate linear regression analysis, presented in Table 4. The multivariate analyses shown in Table 4 included collinearity statistics with independent variables that had significant BH results, which were not highly collinear (VIF < 2.5). Multivariate analysis was only performed if there were more than three significant independent variables following the BH procedure. The summary of the multivariate analyses in Table 4 is as follows:

Total taste score: The following variables retained a statistically significant negative association with the total taste score following multivariate analysis: age (*p* = 0.001), sex (*p* < 0.001), zinc deficiency (*p* = 0.038), major trauma (*p* < 0.001), and current chemotherapy (*p* < 0.001). The parameter S/P URTI retained its statistically significant positive association with the total taste score (*p* = 0.013) (Table 4).

Sweet and sour score, as can be seen in Table 3, following the BH procedure:

Sweet score retained a statistically significant association only with major trauma (*p* = 0.001).

Sour score retained a statistically significant association only with major trauma (*p* = 0.002) and sex (*p* < 0.001).

Considering that, following the BH analyses, only one significant variable for the sweet score and two variables for the sour were retained, there was no need for multivariate analysis for these scores. Therefore, Table 4 does not include a multivariate analysis for sweet and sour scores.

Salty score. The following variables retained their statistically significant negative association with the salty score following multivariate analysis: sex (*p* = 0.003), zinc deficiency (*p* = 0.027), exposure to toxic chemicals (*p* = 0.024), major trauma (*p* = 0.016), and current chemotherapy (*p* = 0.003). S/P URTI (*p* = 0.005) and BMS (*p* = 0.017) retained a statistically significant positive association with total taste score (Table 4).

Bitter score. The following variables retained their statistically significant negative association with bitter score following multivariate analysis: sex (*p* < 0.001), major trauma (*p* < 0.001), and current chemotherapy (*p* = 0.001) (Table 4).

## 4. Discussion

This study retrospectively analyzed the associations of gustatory dysfunction, as measured by validated taste strips with demographics and co-morbidities over 4 years. The present study identified specific patient demographics and co-morbidities that are associated with taste dysfunction and include older age; male sex; and co-morbidities of major trauma, current chemotherapy, zinc deficiency, BMS, and exposure to toxins.

Age. Older age was associated with worse total taste scores in the multivariate analysis. Our findings are in line with the literature, which has demonstrated that taste perception becomes somewhat impaired with normal aging [11,20]. Barragán et al. also found that increased age was associated with a decrease in the perception of all taste qualities, mainly in bitter and sour tastes [21]. Braun et al. also found a decline in oral sensitivity, taste, and smell in older adults [22]. Age-related changes were attributed to changes in taste cell membranes involving altered function of ion channels and receptors, and loss of taste buds [23,24,25]. Indeed, a previous study demonstrated that receptor cell generation in taste organoids was age-related [26]. In the context of age-related changes in taste, it should be mentioned that olfactory dysfunction occurs during the earliest stages of several neurologic disorders, most notably Alzheimer’s disease and Parkinson’s disease, likely heralding the onset of the underlying pathologies [27]. Pavlidis et al. [28] studied 156 nonsmokers and investigated the age-related changes in electrogustometry (EGM) thresholds, as well as in the morphology and density of the fungiform papillae and in the shape and density of vessels at the tip of the human tongue obtained by use of contact endoscopy. Elderly patients exhibited significantly higher EGM thresholds at the chorda tympani area, lower vascular and fungiform papillae density, and worsened vascular morphology at the tip of the tongue [28]. As global population ageing accelerates in the coming decades, maintaining taste sensations and sensitivity in older adults will be a key measure to ensuring quality of health and life [29].

Sex. Men exhibited worse total, salty, and bitter scores compared with women following multivariate analysis. Other studies also showed that women perceive all tastes more [21]. Moreover, numerous studies confirm that men and women differ in terms of their ability to detect or resolve slight differences in tastant concentration [17,30,31]. In [17], the degree of sex-related taste differences depended upon the tongue region evaluated. Pavlidis et al. demonstrated that the density of fungiform papillae is more susceptible to the aging process in males than fungiform papillae shape and tongue tip vascularization are. They also showed that a strong correlation exists between EGM thresholds and vascular shape at the tongue tip in females, especially those belonging in the age group of 50–60 years [28]. Moreover, in addition to the fact that taste information from the periphery varies between males and females, there is a differential central modulation of taste input based on sex [32].

Major trauma. In this study, major trauma—i.e., head and neck trauma (but not minor trauma, i.e., dental trauma)—was associated with worse taste scores, both in specialized assessments and overall taste evaluations. Despite reports of post-traumatic anosmia dating back to the 1800s, relatively few studies have investigated post-traumatic gustatory deficits using validated evaluation tests [33]. Among patients with previous head trauma, 19% exhibited taste deficits [33]; conversely, among patients with taste disorders, 24% had post-traumatic disorders [34]. The estimated incidence of taste loss after head injuries ranges from 0.40% to 5 [35,36,37]. Gustatory dysfunctions can arise after trauma due to direct injury to the tongue and taste buds, damage to cranial nerves, brain contusion, or hemorrhage, and may involve peripheral and/or central mechanisms [1].

Chemotherapy. In the current research, there were reduced taste scores in the total, salty, and bitter scores in individuals currently undergoing chemotherapy. The types of chemotherapeutic agents and types of cancers for which they were used in this study included Rituximab for Lymphoma (three cases) and Cisplatin for oropharyngeal carcinoma (two cases). These findings are consistent with previous reports demonstrating that ~70–75% of patients experience taste disturbances depending on the specific treatment regimens [38,39,40]. Patient age, oral discomfort, and swallowing difficulty were found to be significant factors for the experienced taste alterations in patients receiving chemotherapy [41].

Zinc deficiency. In this study, patients with zinc deficiency exhibited worse salty and total taste scores. Zinc deficiency has been identified as a causative factor in taste disorders that may lead to appetite changes [42,43]. Zinc plays a crucial role in saliva action, food digestion, and the normal function of taste receptors [6]. Patients with taste disturbances often exhibit lower concentrations of serum zinc [44,45]. Gene–nutrient interactions between the αENaC A663T genotype and zinc intake have been implicated in determining salty taste acuity [46]. A study conducted on mice demonstrated that CuCl_2_ and ZnCl_2_ competitively inhibit the binding of sucrose and other sugars to sweet receptor molecules, aligning with our finding of impaired sweet taste associated with zinc deficiency [47]. Experimental zinc depletion has been shown to decrease salty taste perception, while zinc supplementation improves taste acuity in the elderly and patients with taste disorders [48,49]. Nevertheless, a systematic review by Cochrane found very low-quality evidence that was insufficient to conclude the role of zinc supplements in improving taste acuity [50].

BMS. Patients with BMS showed higher salty scores. A recent study reported significantly increased electrogustometric thresholds in BMS patients on the right side of the dorsum and lateral side of the tongue [51]. Klasser et al. also noted persistent dysgeusia and altered taste perception in individuals with BMS, although the exact mechanism underlying these changes remains unclear, with the possible involvement of peripheral or central pathways [52]. Interestingly, treatment of BMS can significantly decrease pain symptoms, resulting in an improvement in taste function [53]. Differences of the oral microbiome and oral pathology could also affect taste. For example, Solomon et al. demonstrated that the values of salivary parameters (calcium, phosphates, and pH) were significantly higher in patients with a small number of dental lesions, compared with the patients who had multiple carious lesions [54]. Moreover, oral mucosal conditions such as desquamative gingivitis, autoimmune bullous dermatoses, lichen planus, and symptomatic migratory stomatitis patients may avoid spicy foods, sour drinks, and alcohol, which exacerbate the pain [55,56].

URTI. Following URTI, total and salty scores were enhanced. This may indicate that changes, if they occurred, were temporary and resolved after the URTI subsided and until the examination was performed. It is well-documented that taste dysfunction may occur alongside the common cold or influenza, often accompanied by olfactory impairment [1,57]. Dysgeusia and, to a lesser extent, a burning sensation in the oral cavity, are not uncommon during URTI [1,37]. Disorders of the smell and taste are more common among patients with COVID-19 compared to patients with influenza [58]. Indeed, in the case of a SARS-CoV-2 infection, a higher prevalence of olfactory and taste disorders was detected [59]; therefore, these cases were excluded from the present study. Another reason for exclusion of post-COVID taste dysfunctions is the finding that patients with COVID-19 may frequently suffer from neuropathies of peripheral nerves that result from immune mechanisms or neurotoxic side effects of drugs used to treat the symptoms of COVID-19 and, to a lesser extent, from the compression of peripheral nerves due to prolonged bedding on the intensive care unit.

Exposure to toxic chemicals. Patients who were exposed to toxic chemicals exhibited worse salty scores. Metals and metalloids, such as mercury, copper, zinc, chromium, arsenic, and lead, have the potential to induce taste alterations [60]. Zheng et al. also found that cadmium exposure is associated with perceived taste dysfunction [61]. Occupational exposure to industrial chemicals in exposed workers can affect taste, such as in the case of industrial or waste-site exposure to toxic levels of metals [62].

In the present study, there were no statistically significant associations between smoking status and taste scores; however, unfortunately, no data regarding alcohol intake were available for analysis. It should be mentioned that it was previously demonstrated that gustatory deficits exist in alcohol dependence (AD) and Korsakoff Syndrome (KS, a neurological complication of AD) [63].

Strengths and limitations. The major strengths of this study include (1) the relatively large sample size; (2) strict adherence to the protocol and work-up of patients, using comprehensive assessment including validated taste strip tests to all patients; (3) the holistic approach that considers important variables such as demographics, history of trauma, and co-morbidities and strict statistical analysis correcting for multiple comparisons.

Limitations include the cross-sectional study design, which suggests associations between variables and cannot address causality. Another limitation is that for the purpose of analyses of systemic co-morbidities, individual diseases were aggregated to create general groups, which may result in false results regarding individual diseases. While data regarding current smoking habits were analyzed, data on past smoking, which may also affect taste scores, were not available. Although the generalizability of the results may be limited, patients were referred from various clinics throughout the country serving diverse populations.

## 5. Conclusions

In conclusion, the present study identified specific patient demographics, i.e., age and sex, and co-morbidities associated with taste dysfunction. These include major trauma, chemotherapy treatment, and zinc deficiency, which significantly reduced taste perception in both specialized and total scores. Exposure to chemicals was associated with a lower salty recognition score. Several illnesses increased taste perception, such as patients with BMS that show higher salty scores. In addition, following URTI total scores, salty and bitter scores were enhanced, which may indicate recovery. The study highlights the importance of systemic evaluation and quantitive gustatory dysfunction assessment as part of the diagnostic process of patients with subjective complaints of taste disorders. Importantly, preventing taste dysfunctions might be possible in some cases by proper diagnosis and treatment of the underlying co-morbidity. Future longitudinal, prospective, multinational studies are needed to assess the mechanisms behind the findings.

## Figures and Tables

**Table 1:** Demographics and clinical parameters of the study population. IQR: Interquartile range,.

Variable	Mean ± Standard Deviation	Median	IQR	Range
Age (years)	53.56 ± 19.27	56	38–68	18–98
Variable		Frequency		Percent
Sex	Men	135		49.6
	Women	137		50.4
Smoking		42		15.4
Trauma	Major trauma	48		17.6
	Minor trauma	16		5.9
S/P Status post surgical procedure		22		8.1
Xerostomia		37		13.6
Hyposalivation		28		10.3
Burning mouth syndrome		16		5.9
Vitamins/nutrient deficiencies	Zinc deficiency	5		1.8
	B12 deficiency	3		1.1
	Iron deficiency	1		0.4
Exposures	Medication-related disturbance disorder	30		11.1
	Exposure to toxic chemicals	3		1.1
	Current chemotherapy treatment	5		1.8
	Past chemotherapy treatment	5		1.8
	Radiotherapy	7		2.6
Current oncologic disease		3		1.1
Past oncologic disease		15		5.5
Hypertension		43		15.8
Diabetes mellitus		37		13.6
Status/post (S/P) Upper respiratory infection (URTI)		37		13.6
Gastrointestinal disease		35		12.9
Cardiovascular disease		27		9.9
Hypothyroidism		26		9.6
Hyperlipidemia		25		9.2
Pulmonary disease		24		8.8
Behavior and psychiatric disorders		24		8.8
Allergic reaction		23		8.5
Neurologic disease		23		8.5
Autoimmune disease		21		7.7
Osteoporosis		10		3.7
Red blood cell disease		10		3.7
Kidney disease		8		2.9
Fibromyalgia		8		2.9
Lichen planus		7		2.6
Obesity (body mass index (BMI) > 30)		5		1.8
Sjögren’s syndrome		4		1.5
Acquired bleeding and hypercoagulable disorder		4		1.5
Liver disease		2		0.7
Deafness		1		0.4
Other		15		5.5

**Table 2:** Statistically significant associations of demographics and co-morbidities with the specific taste and the total taste scores. * Pearson’s correlation, ** Independent *t*-test, SD: standard deviation, S/P: status post, S/P URTI: S/P upper respiratory tract infection, BMS: burning mouth syndrome (*p* > 0.05).

		Sweet Score (Mean ± SD)	Sour Score (Mean ± SD)	Salty Score (Mean ± SD)	Bitter Score (Mean ± SD)	Total Taste Score (Mean ± SD)
Age	Correlation Coefficient	−0.106	−0.141	−0.074	−0.139	−0.156
	*p*-value *	0.099	0.027	0.251	0.030	0.013
Sex	Women	2.74 ± 1.17	2.23 ± 1.04	2.38 ± 1.22	2.66 ± 1.25	9.98 ± 3.59
	Men	2.31 ± 1.39	1.60 ± 1.04	1.79 ± 1.27	1.51 ± 1.36	7.07 ± 3.93
	*p*-value **	0.009	<0.001	<0.001	<0.001	<0.001
Hyposalivation	No	2.59 ± 1.28	1.93 ± 1.11	2.10 ± 1.27	2.08 ± 1.44	8.62 ± 4.06
	Yes	2.03 ± 1.4	1.77 ± 0.89	2.0 ± 1.35	2.11 ± 1.31	7.85 ± 3.76
	*p*-value **	0.038	0.473	0.693	0.936	0.351
BMS	No	2.52 ± 1.32	1.89 ± 1.09	2.03 ± 1.29	2.05 ± 1.439	8.39 ± 4.09
	Yes	2.93 ± 0.79	2.33 ± 0.97	2.93 ± 0.79	2.6 ± 1.12	10.68 ± 1.99
	*p*-value **	0.216	0.130	0.009	0.153	0.027
Major trauma	No	2.66 ± 1.22	2.02 ± 1.06	2.20 ± 1.26	2.27 ± 1.37	9.06 ± 3.81
	Yes	1.87 ± 1.47	1.44 ± 1.07	1.55 ± 1.25	1.20 ± 1.35	6.01 ± 4.15
	*p*-value **	<0.001	0.001	0.002	<0.001	<0.001
Minor trauma	No	2.55 ± 1.30	1.91 ± 1.10	2.11 ± 1.29	2.07 ± 1.44	8.57 ± 4.06
	Yes	2.03 ± 1.21	2.00 ± 0.93	1.69 ± 1.10	2.30 ± 1.03	7.88 ± 3.61
	*p*-value **	0.164	0.787	0.249	0.573	0.55
Zinc deficiency	No	2.55 ± 1.29	1.93 ± 1.09	2.11 ± 1.27	2.10 ± 1.42	8.62 ± 4.02
	Yes	1.20 ± 0.83	1.20 ± 0.83	0.80 ± 0.83	1.20 ± 1.09	4.40 ± 2.96
	*p*-value **	0.021	0.136	0.023	0.159	0.02
Exposure to toxic chemicals	No	2.53 ± 1.29	1.93 ± 1.08	2.11 ± 1.27	2.10 ± 1.42	8.58 ± 4.02
	Yes	2.00 ± 2.00	1.00 ± 1.00	0.33 ± 0.57	1.00 ± 1.00	4.33 ± 3.21
	*p*-value **	0.481	0.142	0.017	0.183	0.069
S/P URTI	No	2.45 ± 1.32	1.86 ± 1.10	1.99 ± 1.28	2.02 ± 1.41	8.24 ± 4.06
	Yes	3.03 ± 1.04	2.32 ± 0.94	2.77 ± 1.05	2.54 ± 1.43	10.56 ± 3.16
	*p*-value **	0.021	0.028	0.001	0.0055	0.002
Gastrointestinal disease (GI)	No	3.49 ± 1.32	1.91 ± 1.09	2.02 ± 1.28	2.03 ± 1.44	8.06 ± 4.09
	Yes	2.74 ± 1.18	1.93 ± 1.10	2.53 ± 1.19	2.45 ± 1.22	9.64 ± 3.47
	*p*-value **	0.313	0.913	0.035	0.115	0.085
Kidney disease	No	2.52 ± 1.31	1.92 ± 1.08	2.08 ± 1.27	2.12 ± 1.42	8.55 ± 4.06
	Yes	2.71 ± 1.11	1.85 ± 1.34	2.28 ± 1.49	1.00 ± 1.00	8.00 ± 3.02
	*p*-value **	0.703	0.877	0.687	0.04	0.702
Obesity	No	2.53 ± 1.29	1.92 ± 1.07	2.09 ± 1.28	2.11 ± 1.42	8.55 ± 4.02
	Yes	2.33 ± 2.08	1.33 ± 2.30	2.00 ± 1.73	0.33 ± 0.57	7.50 ± 5.25
	*p*-value **	0.795	0.35	0.901	0.031	0.605
Autoimmune disease	No	2.58 ± 1.27	1.93 ± 1.07	2.10 ± 1.29	2.10 ± 1.44	8.61 ± 4.04
	Yes	1.82 ± 1.50	1.70 ± 1.26	1.88 ± 1.16	1.88 ± 1.11	7.65 ± 3.84
	*p*-value **	0.021	0.402	0.486	0.534	0.306
Current chemotherapy treatment	No	2.55 ± 1.29	1.94 ± 1.08	2.12 ± 1.27	2.12 ± 1.41	8.64 ± 3.99
	Yes	1.4 ± 1.14	0.8 ± 0.83	0.6 ± 0.54	0.4 ± 0.54	3.2 ± 2.28
	*p*-value **	0.05	0.02	0.008	0.007	0.003

**Table 3:** Benjamini–Hochberg (BH) procedure to decrease the false discovery rate (FDR). GI: Gastrointestinal disease, BMS: burning mouth syndrome, S/P URTI: S/P upper respiratory tract infection.

Taste Score	Variable	Corrected *p*-Value	i	*p* Value Level for FDR	Number of Comparisons	Crit	Test
Total taste score	Kidney disease	0.702	14	0.05	14	0.05	Not Significant
	obesity	0.605	13	0.05	14	0.046429	Not Significant
	Minor trauma	0.55	12	0.05	14	0.042857	Not Significant
	Hyposalivation	0.351	11	0.05	14	0.039286	Not Significant
	Autoimmune disease	0.306	10	0.05	14	0.035714	Not Significant
	GI	0.085	9	0.05	14	0.032143	Not Significant
	Exposure to toxins	0.069	8	0.05	14	0.028571	Not Significant
	BMS	0.027	7	0.05	14	0.025	Not Significant
	Zinc deficiency	0.02	6	0.05	14	0.021429	Significant
	Age	0.013	5	0.05	14	0.017857	Significant
	Current chemotherapy	0.003	4	0.05	14	0.014286	Significant
	S/P URTI	0.002	3	0.05	14	0.010714	Significant
	Major trauma	0	2	0.05	14	0.007143	Significant
	Sex	0	1	0.05	14	0.003571	Significant
Sweet score	Age	0.909	14	0.05	14	0.05	Not Significant
	obesity	0.795	13	0.05	14	0.046429	Not Significant
	Kidney disease	0.703	12	0.05	14	0.042857	Not Significant
	Exposure to toxins	0.481	11	0.05	14	0.039286	Not Significant
	GI	0.313	10	0.05	14	0.035714	Not Significant
	BMS	0.216	9	0.05	14	0.032143	Not Significant
	Minor trauma	0.164	8	0.05	14	0.028571	Not Significant
	Current chemotherapy	0.05	7	0.05	14	0.025	Not Significant
	Hyposalivation	0.038	6	0.05	14	0.021429	Not Significant
	Autoimmune disease	0.021	5	0.05	14	0.017857	Not Significant
	S/P URTI	0.021	4	0.05	14	0.014286	Not Significant
	Zinc deficiency	0.021	3	0.05	14	0.010714	Not Significant
	Sex	0.009	2	0.05	14	0.007143	Not Significant
	Major trauma	0.001	1	0.05	14	0.003571	Significant
Sour score	GI	0.913	14	0.05	14	0.05	Not Significant
	Kidney disease	0.877	13	0.05	14	0.046429	Not Significant
	Minor trauma	0.787	12	0.05	14	0.042857	Not Significant
	Hyposalivation	0.473	11	0.05	14	0.039286	Not Significant
	Autoimmune disease	0.402	10	0.05	14	0.035714	Not Significant
	obesity	0.35	9	0.05	14	0.032143	Not Significant
	Exposure to toxins	0.142	8	0.05	14	0.028571	Not Significant
	Zinc deficiency	0.136	7	0.05	14	0.025	Not Significant
	BMS	0.13	6	0.05	14	0.021429	Not Significant
	S/P URTI	0.028	5	0.05	14	0.017857	Not Significant
	Age	0.027	4	0.05	14	0.014286	Not Significant
	Current chemotherapy	0.02	3	0.05	14	0.010714	Not Significant
	Major trauma	0.002	2	0.05	14	0.007143	Significant
	Sex	0	1	0.05	14	0.003571	Significant
Salty score	obesity	0.901	14	0.05	14	0.05	Not Significant
	Hyposalivation	0.693	13	0.05	14	0.046429	Not Significant
	Kidney disease	0.687	12	0.05	14	0.042857	Not Significant
	Autoimmune disease	0.486	11	0.05	14	0.039286	Not Significant
	Age	0.251	10	0.05	14	0.035714	Not Significant
	Minor trauma	0.249	9	0.05	14	0.032143	Not Significant
	GI	0.035	8	0.05	14	0.028571	Not Significant
	Zinc deficiency	0.023	7	0.05	14	0.025	Significant
	Exposure to toxins	0.017	6	0.05	14	0.021429	Significant
	BMS	0.009	5	0.05	14	0.017857	Significant
	Current chemotherapy	0.008	4	0.05	14	0.014286	Significant
	S/P URTI	0.001	3	0.05	14	0.010714	Significant
	Major trauma	0	2	0.05	14	0.007143	Significant
	Sex	0	1	0.05	14	0.003571	Significant
Bitter score	Hyposalivation	0.936	14	0.05	14	0.05	Not Significant
	Minor trauma	0.573	13	0.05	14	0.046429	Not Significant
	Autoimmune disease	0.534	12	0.05	14	0.042857	Not Significant
	Exposure to toxins	0.183	11	0.05	14	0.039286	Not Significant
	Zinc deficiency	0.159	10	0.05	14	0.035714	Not Significant
	BMS	0.153	9	0.05	14	0.032143	Not Significant
	GI	0.115	8	0.05	14	0.028571	Not Significant
	Kidney disease	0.04	7	0.05	14	0.025	Not Significant
	obesity	0.031	6	0.05	14	0.021429	Not Significant
	Age	0.03	5	0.05	14	0.017857	Not Significant
	Current chemotherapy	0.007	4	0.05	14	0.014286	Significant
	S/P URTI	0.0055	3	0.05	14	0.010714	Significant
	Major trauma	0	2	0.05	14	0.007143	Significant
	Sex	0	1	0.05	14	0.003571	Significant

**Table 4:** Multivariate linear regression analysis including collinearity statistics with the specific taste and the total taste scores. VIF: variance inflation factor, Std. Error: standard error, sig: significance, BMS: burning mouth syndrome, S/P URTI: S/P upper respiratory tract infection.

		Unstandardized Coefficients		Standardized Coefficients	t	Sig.	95.0% Confidence Interval for B		Collinearity Statistics	
		B	Std. Error	Beta			Lower Bound	Upper Bound	Tolerance	VIF
Total taste score	(Constant)	15.157	0.947		16.004	<0.001	13.291	17.023		
	Zinc deficiency	−3.252	1.559	−0.113	−2.085	0.038	−6.323	−0.180	0.972	1.029
	Age	−0.038	0.011	−0.181	−3.284	0.001	−0.060	−0.015	0.946	1.058
	Current chemotherapy	−5.984	1.545	−0.209	−3.874	<0.001	−9.027	−2.941	0.990	1.010
	URTI	1.639	0.658	0.136	2.490	0.013	0.342	2.936	0.958	1.044
	Major trauma	−2.797	0.608	−0.260	−4.598	<0.001	−3.995	−1.598	0.896	1.116
	Sex	−2.736	0.439	−0.340	−6.235	<0.001	−3.601	−1.872	0.965	1.036
Salty score	(Constant)	2.823	0.242		11.689	<0.001	2.347	3.299		
	Zinc deficiency	−1.186	0.533	−0.131	−2.222	0.027	−2.237	−0.135	0.976	1.025
	Exposure to toxic chemicals	−1.559	0.686	−0.134	−2.274	0.024	−2.910	−0.208	0.976	1.024
	BMS	0.762	0.317	0.143	2.405	0.017	0.138	1.386	0.963	1.038
	Current chemotherapy	−1.572	0.531	−0.174	−2.961	0.003	−2.617	−0.526	0.986	1.014
	URTI	0.656	0.231	0.171	2.836	0.005	0.200	1.113	0.938	1.066
	Major trauma	−0.497	0.205	−0.148	−2.417	0.016	−0.902	−0.092	0.910	1.099
	Sex	−0.465	0.153	−0.182	−3.042	0.003	−0.766	−0.164	0.953	1.049
Bitter score	(Constant)	3.853	0.253		15.253	<0.001	3.355	4.350		
	Current chemotherapy	−1.950	0.561	−0.194	−3.476	0.001	−3.055	−0.845	0.991	1.009
	URTI	0.323	0.242	0.076	1.335	0.183	−0.154	0.800	0.963	1.038
	Major trauma	−0.871	0.214	−0.233	−4.075	<0.001	−1.292	−0.450	0.943	1.060
	Sex	−1.073	0.160	−0.377	−6.713	<0.001	−1.388	−0.758	0.979	1.022

## Data Availability

The data presented in this study are available on request from the corresponding author. The data are not publicly available due to privacy and ethical restrictions.

## Supplementary Materials

The following supporting information can be downloaded at: https://www.mdpi.com/article/10.3390/biology13010050/s1, Table S1: Sex; Table S2: Hyposalivation; Table S3: BMS; Table S4: Major trauma; Table S5: Minor trauma; Table S6: Zinc deficiency; Table S7: Exposure to toxic chemicals; Table S8: S/P URTI; Table S9: Gastrointestinal disease; Table S10: Kidney disease; Table S11: Obesity; Table S12: Immune-related disease; Table S13: Current chemotherapy.
