# Supplementary material for: Quantitive Assessment of Gustatory Function and Its Association with Demographics, and Systemic Morbidity
Source: Biology (Basel). 2024 Jan 18;13(1):50. doi: 10.3390/biology13010050 (PMC10813619; doi:10.3390/biology13010050)
Supplement: Supplementary file 1 [file biology-13-00050-s001.zip › biology-2756420-supplementary.pdf]

Supplementary file

Nonparametric tests for the different variables: Independent-Samples Mann-Whitney U Test, Kruskal Wallis and spearman's Rho

Table S1: Sex.

Hypothesis Test Summary

	Null Hypothesis	Test	Sig.	Decision
1	The distribution of Sweet is the same across categories of Sex.	Independent-Samples Mann-Whitney U Test	.019	Reject the null hypothesis.
2	The distribution of Sour is the same across categories of Sex.	Independent-Samples Mann-Whitney U Test	.000	Reject the null hypothesis.
3	The distribution of Salty is the same across categories of Sex.	Independent-Samples Mann-Whitney U Test	.000	Reject the null hypothesis.
4	The distribution of Bitter is the same across categories of Sex.	Independent-Samples Mann-Whitney U Test	.000	Reject the null hypothesis.
5	The distribution of Total taste score is the same across categories of Sex.	Independent-Samples Mann-Whitney U Test	.000	Reject the null hypothesis.

Asymptotic significances are displayed. The significance level is .050.

Table S2: Hyposalivation

Hypothesis Test Summary

	Null Hypothesis	Test	Sig.	Decision
1	The distribution of Sweet is the same across categories of Hyposalivation.	Independent-Samples Mann-Whitney U Test	.044	Reject the null hypothesis.
2	The distribution of Sour is the same across categories of Hyposalivation.	Independent-Samples Mann-Whitney U Test	.412	Retain the null hypothesis.
3	The distribution of Salty is the same across categories of Hyposalivation.	Independent-Samples Mann-Whitney U Test	.616	Retain the null hypothesis.
4	The distribution of Bitter is the same across categories of Hyposalivation.	Independent-Samples Mann-Whitney U Test	.987	Retain the null hypothesis.
5	The distribution of Total taste score is the same across categories of Hyposalivation.	Independent-Samples Mann-Whitney U Test	.310	Retain the null hypothesis.

Asymptotic significances are displayed. The significance level is .050.

Table S3: BMS

Hypothesis Test Summary

	Null Hypothesis	Test	Sig.	Decision
1	The distribution of Sweet is the same across categories of Burning mouth syndrome (BMS).	Independent-Samples Mann-Whitney U Test	.385	Retain the null hypothesis.
2	The distribution of Sour is the same across categories of Burning mouth syndrome (BMS).	Independent-Samples Mann-Whitney U Test	.180	Retain the null hypothesis.
3	The distribution of Salty is the same across categories of Burning mouth syndrome (BMS).	Independent-Samples Mann-Whitney U Test	.009	Reject the null hypothesis.
4	The distribution of Bitter is the same across categories of Burning mouth syndrome (BMS).	Independent-Samples Mann-Whitney U Test	.170	Retain the null hypothesis.
5	The distribution of Total taste score is the same across categories of Burning mouth syndrome (BMS).	Independent-Samples Mann-Whitney U Test	.032	Reject the null hypothesis.

Asymptotic significances are displayed. The significance level is .050.

Table S4: Major trauma

Hypothesis Test Summary

	Null Hypothesis	Test	Sig.	Decision
1	The distribution of Sweet is the same across categories of Major trauma.	Independent-Samples Mann-Whitney U Test	.001	Reject the null hypothesis.
2	The distribution of Sour is the same across categories of Major trauma.	Independent-Samples Mann-Whitney U Test	.001	Reject the null hypothesis.
3	The distribution of Salty is the same across categories of Major trauma.	Independent-Samples Mann-Whitney U Test	.004	Reject the null hypothesis.
4	The distribution of Bitter is the same across categories of Major trauma.	Independent-Samples Mann-Whitney U Test	.000	Reject the null hypothesis.
5	The distribution of Total taste score is the same across categories of Major trauma.	Independent-Samples Mann-Whitney U Test	.000	Reject the null hypothesis.

Asymptotic significances are displayed. The significance level is .050.

Table S5: Minor trauma:

Hypothesis Test Summary

	Null Hypothesis	Test	Sig.	Decision
1	The distribution of Sweet is the same across categories of Minor trauma.	Independent-Samples Mann-Whitney U Test	.132	Retain the null hypothesis.
2	The distribution of Sour is the same across categories of Minor trauma.	Independent-Samples Mann-Whitney U Test	.775	Retain the null hypothesis.
3	The distribution of Salty is the same across categories of Minor trauma.	Independent-Samples Mann-Whitney U Test	.209	Retain the null hypothesis.
4	The distribution of Bitter is the same across categories of Minor trauma.	Independent-Samples Mann-Whitney U Test	.644	Retain the null hypothesis.
5	The distribution of Total taste score is the same across categories of Minor trauma.	Independent-Samples Mann-Whitney U Test	.481	Retain the null hypothesis.

Asymptotic significances are displayed. The significance level is .050.

Table S6: Zinc deficiency

Hypothesis Test Summary

	Null Hypothesis	Test	Sig.	Decision
1	The distribution of Sweet is the same across categories of Zinc deficiency.	Independent-Samples Mann-Whitney U Test	.018	Reject the null hypothesis.
2	The distribution of Sour is the same across categories of Zinc deficiency.	Independent-Samples Mann-Whitney U Test	.119	Retain the null hypothesis.
3	The distribution of Salty is the same across categories of Zinc deficiency.	Independent-Samples Mann-Whitney U Test	.023	Reject the null hypothesis.
4	The distribution of Bitter is the same across categories of Zinc deficiency.	Independent-Samples Mann-Whitney U Test	.158	Retain the null hypothesis.
5	The distribution of Total taste score is the same across categories of Zinc deficiency.	Independent-Samples Mann-Whitney U Test	.023	Reject the null hypothesis.

Asymptotic significances are displayed. The significance level is .050.

Table S7: <u>Exposure to toxic chemicals</u>

Hypothesis Test Summary

	Null Hypothesis	Test	Sig.	Decision
1	The distribution of Sweet is the same across categories of Exposure to toxic chemicals.	Independent-Samples Mann-Whitney U Test	.584	Retain the null hypothesis.
2	The distribution of Sour is the same across categories of Exposure to toxic chemicals.	Independent-Samples Mann-Whitney U Test	.144	Retain the null hypothesis.
3	The distribution of Salty is the same across categories of Exposure to toxic chemicals.	Independent-Samples Mann-Whitney U Test	.022	Reject the null hypothesis.
4	The distribution of Bitter is the same across categories of Exposure to toxic chemicals.	Independent-Samples Mann-Whitney U Test	.177	Retain the null hypothesis.
5	The distribution of Total taste score is the same across categories of Exposure to toxic chemicals.	Independent-Samples Mann-Whitney U Test	.073	Retain the null hypothesis.

Asymptotic significances are displayed. The significance level is .050.

Table S8: **S/P URTI**

Hypothesis Test Summary

	Null Hypothesis	Test	Sig.	Decision
1	The distribution of Sweet is the same across categories of Upper respiratory tract infection (URTI).	Independent-Samples Mann-Whitney U Test	.022	Reject the null hypothesis.
2	The distribution of Sour is the same across categories of Upper respiratory tract infection (URTI).	Independent-Samples Mann-Whitney U Test	.027	Reject the null hypothesis.
3	The distribution of Salty is the same across categories of Upper respiratory tract infection (URTI).	Independent-Samples Mann-Whitney U Test	.002	Reject the null hypothesis.
4	The distribution of Bitter is the same across categories of Upper respiratory tract infection (URTI).	Independent-Samples Mann-Whitney U Test	.049	Reject the null hypothesis.
5	The distribution of Total taste score is the same across categories of Upper respiratory tract infection (URTI).	Independent-Samples Mann-Whitney U Test	.003	Reject the null hypothesis.

Asymptotic significances are displayed. The significance level is .050.

Table S9: **Gastrointestinal disease**

Hypothesis Test Summary

	Null Hypothesis	Test	Sig.	Decision
1	The distribution of Sweet is the same across categories of Gastrointestinal disease .	Independent-Samples Mann-Whitney U Test	.351	Retain the null hypothesis.
2	The distribution of Sour is the same across categories of Gastrointestinal disease .	Independent-Samples Mann-Whitney U Test	.955	Retain the null hypothesis.
3	The distribution of Salty is the same across categories of Gastrointestinal disease .	Independent-Samples Mann-Whitney U Test	.026	Reject the null hypothesis.
4	The distribution of Bitter is the same across categories of Gastrointestinal disease.	Independent-Samples Mann-Whitney U Test	.128	Retain the null hypothesis.
5	The distribution of Total taste score is the same across categories of Gastrointestinal disease.	Independent-Samples Mann-Whitney U Test	.087	Retain the null hypothesis.

Asymptotic significances are displayed. The significance level is .050.

Table S10: Kidney disease

Hypothesis Test Summary

	Null Hypothesis	Test	Sig.	Decision
1	The distribution of Sweet is the same across categories of Kidney disease.	Independent-Samples Mann-Whitney U Test	.825	Retain the null hypothesis.
2	The distribution of Sour is the same across categories of Kidney disease.	Independent-Samples Mann-Whitney U Test	.802	Retain the null hypothesis.
3	The distribution of Salty is the same across categories of Kidney disease.	Independent-Samples Mann-Whitney U Test	.695	Retain the null hypothesis.
4	The distribution of Bitter is the same across categories of Kidney disease.	Independent-Samples Mann-Whitney U Test	.041	Reject the null hypothesis.
5	The distribution of Total taste score is the same across categories of Kidney disease.	Independent-Samples Mann-Whitney U Test	.560	Retain the null hypothesis.

Asymptotic significances are displayed. The significance level is .050.

Table S11: Obesity:

Hypothesis Test Summary

	Null Hypothesis	Test	Sig.	Decision
1	The distribution of Sweet is the same across categories of Obesity.	Independent-Samples Mann-Whitney U Test	.970	Retain the null hypothesis.
2	The distribution of Sour is the same across categories of Obesity.	Independent-Samples Mann-Whitney U Test	.399	Retain the null hypothesis.
3	The distribution of Salty is the same across categories of Obesity.	Independent-Samples Mann-Whitney U Test	.997	Retain the null hypothesis.
4	The distribution of Bitter is the same across categories of Obesity.	Independent-Samples Mann-Whitney U Test	.036	Reject the null hypothesis.
5	The distribution of Total taste score is the same across categories of Obesity.	Independent-Samples Mann-Whitney U Test	.699	Retain the null hypothesis.

Asymptotic significances are displayed. The significance level is .050.

Table S12: Immune-related disease

Hypothesis Test Summary

	Null Hypothesis	Test	Sig.	Decision
1	The distribution of Sweet is the same across categories of Immune-related disease.	Independent-Samples Mann-Whitney U Test	.036	Reject the null hypothesis.
2	The distribution of Sour is the same across categories of Immune-related diseases.	Independent-Samples Mann-Whitney U Test	.548	Retain the null hypothesis.
3	The distribution of Salty is the same across categories of Immune-related diseases.	Independent-Samples Mann-Whitney U Test	.463	Retain the null hypothesis.
4	The distribution of Bitter is the same across categories of Immune-related disease.	Independent-Samples Mann-Whitney U Test	.471	Retain the null hypothesis.
5	The distribution of Total taste score is the same across categories of Immune-related diseases.	Independent-Samples Mann-Whitney U Test	.270	Retain the null hypothesis.

Asymptotic significances are displayed. The significance level is .050.

Table S13: Current chemotherapy

Hypothesis Test Summary

	Null Hypothesis	Test	Sig.	Decision
1	The distribution of Sweet is the same across categories of Current chemotherapy treatment.	Independent-Samples Mann-Whitney U Test	.049	Reject the null hypothesis.
2	The distribution of Sour is the same across categories of Current chemotherapy treatment.	Independent-Samples Mann-Whitney U Test	.023	Reject the null hypothesis.
3	The distribution of salt is the same across categories of Current chemotherapy treatment.	Independent-Samples Mann-Whitney U Test	.009	Reject the null hypothesis.
4	The distribution of Bitter is the same across categories of Current chemotherapy treatment.	Independent-Samples Mann-Whitney U Test	.009	Reject the null hypothesis.
5	The distribution of Total taste score is the same across categories of Current chemotherapy treatment.	Independent-Samples Mann-Whitney U Test	.005	Reject the null hypothesis.

Asymptotic significances are displayed. The significance level is .050.
